# Murine Mesenchymal Stromal Cells Retain Biased Differentiation Plasticity Towards Their Tissue of Origin

**DOI:** 10.3390/cells9030756

**Published:** 2020-03-19

**Authors:** Ting Ting Ng, Kylie Hin-Man Mak, Christian Popp, Ray Kit Ng

**Affiliations:** 1 School of Biomedical Sciences, Li Ka Shing Faculty of Medicine, The University of Hong Kong, Hong Kong SAR, China; 2 Shenzhen Institute of Research and Innovation, The University of Hong Kong, Hong Kong SAR, China

**Keywords:** mesenchymal stromal cell, differentiation, tissue of origin, prolonged culture, epigenetic memory

## Abstract

Mesenchymal stromal/stem cells (MSCs) reside in many human tissues and comprise a heterogeneous population of cells with self-renewal and multi-lineage differentiation potential, making them useful in regenerative medicine. It remains inconclusive whether MSCs isolated from different tissue sources exhibit variations in biological features. In this study, we derived MSCs from adipose tissue (AT-MSC) and compact bone (CB-MSC). We found that early passage of MSCs was readily expandable ex vivo, whereas the prolonged culture of MSCs showed alteration of cell morphology to fibroblastoid and reduced proliferation. CB-MSCs and AT-MSCs at passage 3 were CD29^+^, CD44^+^, CD105^+^, CD106^+^, and Sca-1^+^; however, passage 7 MSCs showed a reduction of MSC markers, indicating loss of stem cell population after prolonged culturing. Strikingly, CB-MSC was found more efficient at undergoing osteogenic differentiation, while AT-MSC was more efficient to differentiate into adipocytes. The biased differentiation pattern of MSCs from adipogenic or osteogenic tissue source was accompanied by preferential expression of the corresponding lineage marker genes. Interestingly, CB-MSCs treated with DNA demethylation agent 5-azacytidine showed enhanced osteogenic and adipogenic differentiation, whereas the treated AT-MSCs are less competent to differentiate. Our results suggest that the epigenetic state of MSCs is associated with the biased differentiation plasticity towards its tissue of origin, proposing a mechanism related to the retention of epigenetic memory. These findings facilitate the selection of optimal tissue sources of MSCs and the ex vivo expansion period for therapeutic applications.

## 1. Introduction

Mesenchymal stromal cells (MSCs), also referred to mesenchymal stem cells [1], represent a heterogeneous population of cells that can be isolated from a wide range of tissues, including bone marrow, compact bone, placenta and adipose tissue [2,3,4,5,6]. MSC was first isolated from mouse bone marrow as fibroblast colony-forming units, which were distinguished by their ability to adhere to plastic culture dishes [2]. They display fibroblastic morphology and are capable of differentiation to chondrocytes, adipocytes, and osteoblasts in vitro [1,7]. Differentiation to other non-mesodermal cell types, such as neurons, muscles, endothelial cells, and hepatocytes, has also been reported [8,9,10,11]. MSCs are intensely studied in clinical research because of their multi-lineage potential and ease of isolation and culture [1,12]. In addition, their ability to evade the host immune system by suppressing T cells, B cells, and natural killer cells [13], and releasing anti-inflammatory proteins [14,15] have made them an important tool for disease treatment. Clinical trials using MSCs for the treatment of osteoarthritis, degenerative disc disease, ischemic heart disease, and stroke are currently undergoing to explore their therapeutic applications [12].

Harvesting MSCs from the non-bone marrow tissue sources can be done by less invasive methods and the primary isolated MSCs can be expanded ex vivo to yield a larger number. Therefore, these non-bone marrow-derived MSCs are considered as an attractive repertoire for stem cell and regenerative medicine. In light of the broad potentials for therapeutic applications and the variety of sources for MSCs, it has been reported that MSCs, regardless of their tissue of origins, displayed similar characteristics in the differentiation to adipocytes, chondrocytes and osteocytes [16,17,18]. However, other studies comparing MSCs from human bone marrow, skin and adipose tissues showed considerable differences in their growth rate and differentiation potentials [19,20], supporting the hypothesis of preferential differentiation hierarchies [21,22]. It, therefore, remains inconclusive on the characteristics and the differentiation potentials of MSCs obtained from various tissue sources.

With such variability in mind, a better understanding of the differences between MSCs from different tissue origins can help identify the most suitable cell source for specific clinical purposes. Here we compared and characterized murine MSCs obtained from the adipose tissues (AT-MSC) and compact bone (CB-MSC) using standard isolation methods and expanded ex vivo at early and late cell passages. Both MSCs cultured for an extended period of time showed morphological changes and a decline in cell proliferation. We also demonstrated the tissue origin of MSC is associated with the alterations of cell surface marker patterns and differentiation potential towards osteogenic and adipogenic lineages. Removal of DNA methylation by pharmacological agent can alter the biased differentiation potential of MSCs dependent on the tissue source.

## 2. Materials and Methods

### 2.1. MSC Isolation and Culture

MSCs were harvested from 8-week old C57BL/6 mice (Laboratory Animal Unit, The University of Hong Kong). Written informed consent to use the animals was approved by the Committee on the Use of Live Animals in Teaching and Research of the University of Hong Kong (Reference no.: 2416-11). Three mice were used for the MSC isolation experiment and a total of five experiments were performed. The male to female ratio was 2:1. Mice were sacrificed by over-dosage of isoflurane inhalation. The MSC isolation procedures were described previously [5,23]. For CB-MSC isolation, muscles from femur, humerus, and tibia were removed. The epiphyseal ends of the bone were cut and discarded. Bone marrow was released by gently crushing the bones in cold PBS with 2% FBS (Gibco, Invitrogen, Grand Island, NY, USA) and 1 mM EDTA (Sigma-Aldrich, St. Louis, MO, USA). The cleaned bone fragments were digested in 0.25% collagenase I (Gibco) with 20% FBS for 5 min at 37 °C. The bone fragments were further chopped into 1–2 mm bits and digested for another 45 min at 37 °C. CB-MSCs were separated from the bone fragments by filtering the cell suspension through a 70μm cell strainer (BD Biosciences, Franklin Lakes, NJ, USA). For AT-MSC isolation, adipose tissue dissected from inguinal and subcutaneous sites was digested in 5% collagenase I (Gibco) for 1 h at 37 °C. Cells were released from the adipose tissue by centrifuging for 5 min at 500× *g*. The released cells were treated with ammonium-chloride-potassium (ACK) lysing buffer (Sigma-Aldrich) for 3 min at room temperature to lyse red blood cells. The cell suspension was washed twice with α-MEM and tissue debris was removed by filtering through a 70 μm cell strainer. The isolated cell suspension (5 × 10^6^) were cultured in α-MEM with 10% fetal bovine serum (FBS) (HyClone, Logan, UT, USA) and 1× penicillin-streptomycin-glutamine (Gibco) on a 100 mm culture dish at 37 °C with 5% CO_2_. MSCs were adhered within 48 h. The culture medium was changed every 2 days. MSCs were passaged in a 1:4 ratio when reaching 80% confluence by Accutase (Gibco) for 5 min at 37 °C.

### 2.2. Cell Proliferation and Immunophenotypic Analysis

AT-MSCs and CB-MSCs (2 × 10^4^ cells) were seeded into 6-well plates on day 0. The total number of expanded cells were counted at day 1, 3 and 5. Cell doubling time (DT) was calculated by the following formula: DT = T × ln2/ln × (Xe/Xb), where T is the incubation time in any units; Xb is the cell number at the beginning of the incubation time; Xe is the cell number at the end of the incubation time.

Cultured cells were stained with FITC-conjugated anti-CD29 or anti-c-kit, PE-conjugated anti-CD44, anti-CD45, anti-CD106 or anti-Sca-1, APC-conjugated anti-CD105, and APC-Cy7-conjugated anti-CD11b (all from Biolegend, San Diego, CA, USA) at a concentration of 0.5 μg/mL for 30 min at 4 °C. The corresponding fluorophore-conjugated isotype controls were used for the gating of the positive-stained cells. Immunophenotypic analysis of 5000–10,000 cells of each sample was performed using FACSCanto II flow cytometer (BD Biosciences). The flow cytometry data were analyzed using Flowjo software (Tree Star, Ashland, OR, USA, ver. 10.0.7). Both assays were performed three times with duplicated samples.

### 2.3. MSC Differentiation Assays

MSCs at passage 3 or 7 (6 × 10^4^ cells) were seeded into 24-well plates. One group of MSCs was treated with 0.5 μM 5-azacytidine (5-aza, Sigma-Aldrich) for 48 h prior to differentiation. For osteogenic differentiation, MSCs were differentiated in α-MEM with 10% FBS and StemXVivo Mouse/Rat Osteogenic Supplement (R&D Systems, Minneapolis, MN, USA) for 18 days. Differentiated cells were fixed in 4% formaldehyde (Sigma-Aldrich) for 10 min at room temperature and stained with 2% Alizarin Red solution (Chemicon, Merck Millipore, Billerica, MA, USA) for 15 min at room temperature. For adipogenic differentiation, MSCs were differentiated in α-MEM with 10% FBS and StemXVivo Adipogenic Supplement (R&D Systems) for 14 days. Differentiated cells were fixed in 4% formaldehyde (Sigma-Aldrich) for 10 min at room temperature and stained with 0.5% Oil Red O solution (Sigma-Aldrich) for 15 min at room temperature. For chondrogenic differentiation, MSCs were centrifuged for 5 min at 200× *g* in a 1.5 mL tube and differentiated in DMEM /F-12 with 1× Insulin-Transferrin-Selenium (Gibco) and StemXVivo Human/Mouse Chondrogenic Supplement (R&D Systems) for 21 days. Chondrocyte spheroids were fixed in 4% formaldehyde (Sigma-Aldrich) for 1 h at room temperature and stained with Alcian Blue 8GX solution (Sigma-Aldrich) for 30 min at room temperature. MSCs cultured in the differentiation medium without supplements were served as controls. The differentiation assay was performed three times with duplicated samples.

### 2.4. RNA Extraction and Quantitative RT-PCR (qRT-PCR)

Total RNA was extracted from the differentiated MSCs using MiniBEST Universal RNA Extraction Kit (Takara, Kusatsu, Japan). Genomic DNA eraser column and DNaseI treatment were used to remove genomic DNA. cDNA was synthesized using PrimeScriptTM RT reagent kit with gDNA Eraser (Takara) according to the manufacturer’s protocol. qRT-PCR was performed with the 7900HT Fast Real-Time PCR System (Applied Biosystems, Waltham, MA, USA) using SYBR Premix Ex TaqTM (Takara) with the oligo primers listed in Supplementary Table S1. *Gapdh* and *β-Actin* served as house-keeping genes for normalization of gene expression. All samples were analyzed in triplicate. Three independent experiments were performed and relative gene expression was calculated using 2^−ΔΔCT^ method.

### 2.5. Statistical Analysis

A statistically significant difference was calculated by two-tailed unpaired Student’s *t*-test.

## 3. Results

### 3.1. Ex Vivo Expansion of MSC Isolated from Compact Bone and Adipose Tissue

**Figure 1:**
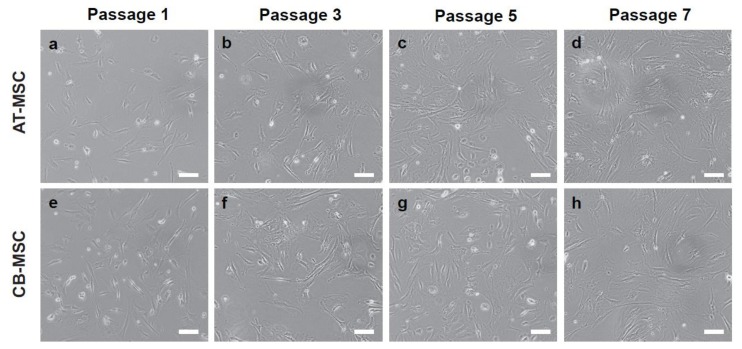
Cell morphology of AT-MSC and CB-MSC. Morphologies of MSCs at (**a**,**e**) passage 1, (**b**,**f**) passage 3, (**c**,**g**) passage 5, and (**d**,**h**) passage 7 were shown. Cell morphology changed gradually from spindle-like to flat and fibroblastoid with increasing passage number. Representative images were taken at 20× magnification. Scale bars: 100 µm.

Murine MSCs isolated from the adipose tissue (AT) and compact bone (CB) were expanded ex vivo. Both AT-MSCs and CB-MSCs displayed spindle-like to fibroblastoid cell morphology [4,5,23]. It was observed that cells at passage one (P1) contain a small number of cells with spherical shape, which were presumably dividing cells or non-MSCs (Figure 1a,e). However, continuous passaging of cells to the third passage (P3), which is one week of culture, gradually eliminated the non-MSC populations and enriched for MSCs (Figure 1b,f). From passage three (P3) onwards, cell morphology changed from elongated to fibroblastoid in both cultures (Figure 1b–d,f–h).

**Figure 2:**
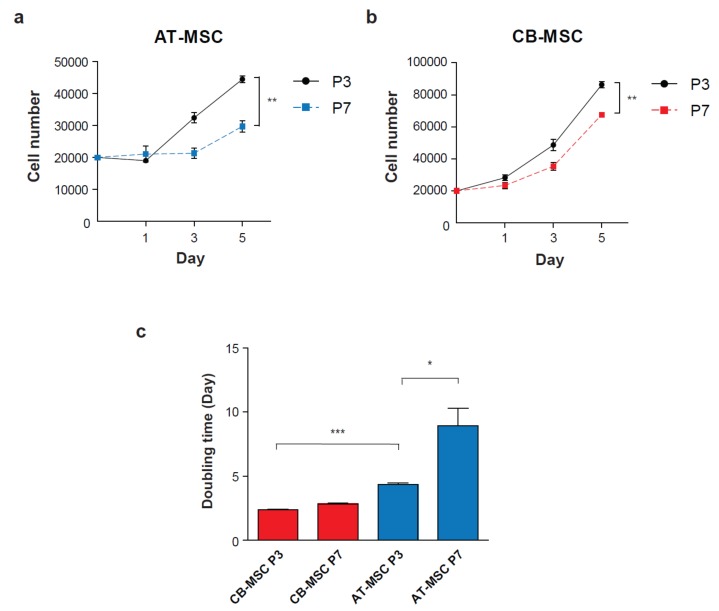
Ex vivo expansion of MSCs at different passage numbers. Cell proliferation assay was performed to determine the growth rate of early (Passage 3) and late passage (Passage 7) of (**a**) AT-MSC and (**b**) CB-MSC. Cells were counted on day 1, 3 and 5 (*n* = 3). (**c**) Doubling times of MSCs were calculated over 5 days of culture. CB-MSCs demonstrated a higher cell proliferation rate than AT-MSCs. The doubling time of AT-MSC was significantly increased at late passage. Experiments were performed with three replicates. Data represent mean ± SD; *p < 0.05, ** *p* < 0.01 and *** *p* < 0.001.

Ex vivo culture of AT-MSC and CB-MSC at P3 or P7 for 5 days demonstrated cell number expansion. P3 and P7 AT-MSC showed limited expansion by 2.2- and 1.5-fold, respectively; whereas P3 and P7 CB-MSC were expanded 4.3- and 3.3-fold, respectively (Figure 2a,b). Besides, it was found that the doubling time of CB-MSC was comparable between both passages (2.4 days and 2.8 days for P4 and P7, respectively); however, AT-MSC demonstrated a significant increase in doubling time from P4 (4.3 days) to P7 (8.9 days) (Figure 2c). These results demonstrated different cell proliferation patterns between MSCs isolated from different tissue origins. Nevertheless, prolonged culture of both types of MSCs gradually reduced proliferation rate beyond passage 7.

### 3.2. Alterations of MSC Immunophenotypes by Prolonged Culture

Previous studies have shown that prolonged culture of MSC altered their immunophenotypes [24]. This prompt us to examine the expression of a panel of mesenchymal stromal cell surface markers, including CD29, CD44, CD105, CD106, and stem cell antigen-1 (Sca-1) [25,26,27,28], in the ex vivo expanded cells. Hematopoietic markers c-kit, CD11b, and CD45 were served as negative markers for the detection of contamination of hematopoietic cells from the MSC isolation procedures [27,29]. c-kit^+^ and CD11b^+^ populations were generally low in both types of MSCs, particularly for the late passage culture (Figure S1). It was observed that 38.4% of CD45^+^ populations were present in P3 CB-MSC, suggesting a low degree of hematopoietic cell contamination from compact bone during MSC isolation.

**Figure 3:**
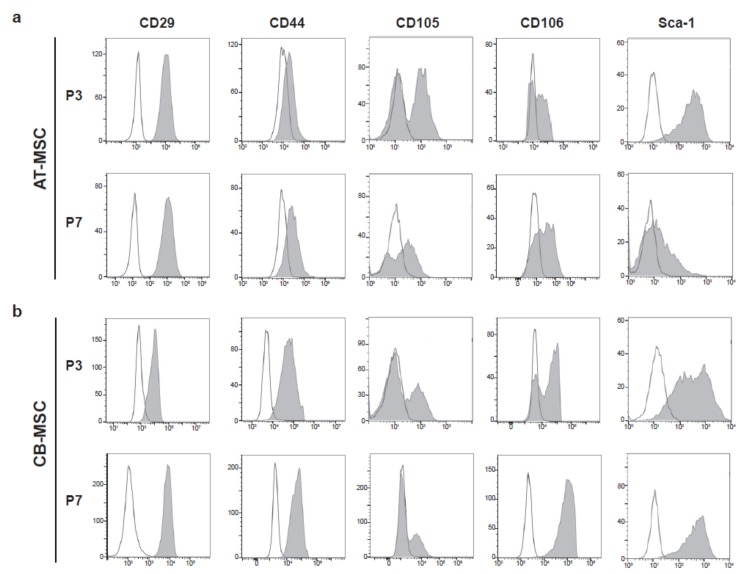
Immunophenotypes of MSCs. Cell surface markers for MSCs, CD29, CD44, CD105, CD106, and Sca-1 were used to characterize (**a**) AT-MSC and (**b**) CB-MSC at passage 3 (P3) and 7 (P7), respectively. Representative flow cytometry patterns were shown. Shaded peaks represent antibody-labeled population; blank peaks represented isotype controls.

Nevertheless, the CD45^+^ hematopoietic cells were gradually lost when cells passaging to P7. Both AT-MSCs and CB-MSCs demonstrated high expression of most of the MSC markers at passage 3. It was noted that CD29^+^, CD44^+^, and CD106^+^ populations showed further increased in passage 7 (Table 1, Figure 3). However, CD105^+^ population was reduced significantly at late passage MSCs. While a significant portion of the AT-MSC population retained as CD105^+^ (33.6 ± 4.3%) at P7, the CD105^+^ population in CB-MSC reduced drastically from 34.2% at P3 to 7.5% at P7. In contrast, CB-MSC consisted of over 83% Sca-1^+^ cells at P3 and P7, whereas the Sca-1^+^ population dropped from 98.5% to 26.3% in AT-MSC from P3 to P7. These immunophenotypic results demonstrated the alteration of MSC surface marker pattern during ex vivo culture, suggesting that prolonged culture of MSC is accompanied by the loss of MSC identity.

### 3.3. Biased Differentiation Towards the Tissue Origin

**Figure 4:**
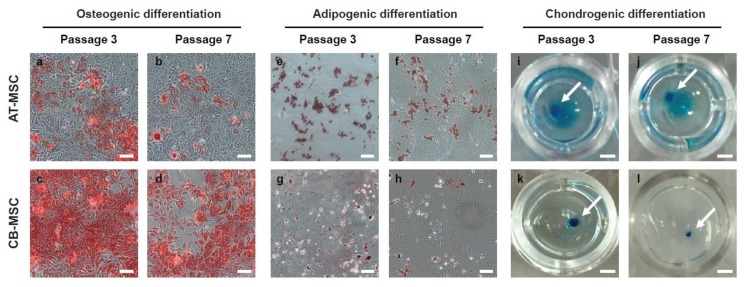
Osteogenic and adipogenic differentiation of MSCs. AT-MSCs and CB-MSCs underwent 18-day of osteogenic, 14-day of adipogenic, or 21-days of chondrogenic differentiation conditions. (**a**–**d**) Alizarin red staining, (**e**–**h**) Oil Red O staining, and (**i**–**l**) Alcian blue staining were used to assess osteogenic, adipogenic and chondrogenic differentiation, respectively. Passage 3 of CB-MSCs displayed stronger staining for Alizarin red; whereas passage 3 of AT-MSCs displayed stronger Oil-Red-O staining. Late passage MSCs showed weaker staining in both lineage differentiations. Chondrogenic differentiation is comparable in both types of MSCs. The white arrows indicate the stained chondrocyte spheroids. Representative images were taken at 20× (**a**–**h**) or 5× (**i**–**l**) magnification. Scale bars: (**a**–**h**) 100 µm; (**i**–**l**) 1 mm.

A defining feature of MSC is their ability to differentiate into multiple mesodermal lineages. To examine the multi-lineage differentiation potentials of MSCs derived from different tissue origins, we induced in vitro differentiation of early and late passage (P3 and P7) AT-MSC and CB-MSC into the osteogenic, adipogenic, and chondrogenic lineages. We observed that both AT-MSCs and CB-MSCs were able to differentiate into the three lineages (Figure 4), indicating that MSCs from adipose tissue or compact bone are multipotent in nature. However, although both types of MSCs were able to form the positive Alcian Blue stained chondrocyte spheroids efficiently (Figure 4i–l), we observed that fewer cells stained positive with Alizarin Red in the AT-MSC sample (Figure 4a–d) and lower number of Oil Red O stained cells from CB-MSC sample (Figure 4e–h). Besides, the P7 MSCs of both types appeared to have weaker positive staining patterns when compared to the early P3 samples. These results suggest that MSCs derived from different tissue origins exhibit differentiation bias and their differentiation capacities reduce after prolonged culture.

**Figure 5:**
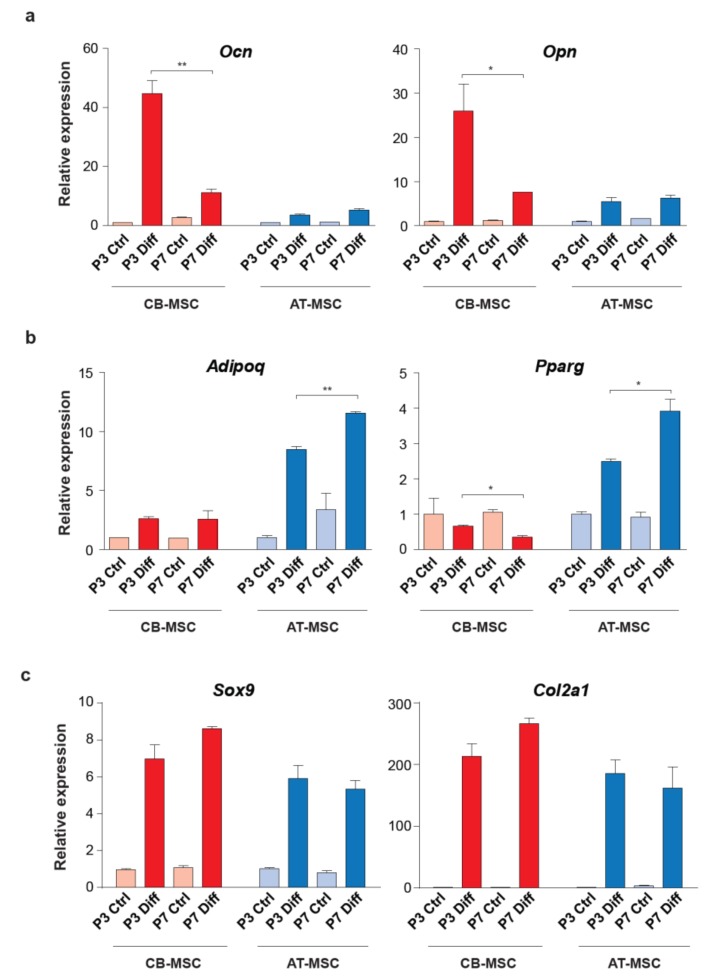
Expression of lineage marker genes in the differentiated MSCs. (**a**) Osteogenic markers, *Ocn* and *Opn*, (**b**) adipogenic markers, *Adipoq* and *Pparg*, and (**c**) chondrogenic markers, *Sox9* and *Col2a1*, were used to determine the multi-lineage differentiation of AT-MSCs and CB-MSCs. MSCs cultured in basic medium without differentiation agents for the same period of time were served as controls. Gene expressions were normalized with housekeeping gene *Gapdh*. Experiments were performed with three replicates. Data represent mean ± SD; *p < 0.05 and ** *p* < 0.01.

To further elucidate the differentiation bias associated with the tissue origin of MSCs, we examined the expression of osteogenic (*Ocn* and *Opn*), adipogenic (*Adipoq* and *Pparg*) and chondrogenic (*Sox9* and *Col2a1*) markers in the differentiated MSC samples. Induction of *Ocn* and *Opn* were high in the osteogenic differentiation of P3 CB-MSC when compared to the P7 CB-MSC (over 4-fold for both genes). Importantly, osteogenic differentiated AT-MSC demonstrated significantly lower expression of these two osteogenic markers, regardless of the length of culture (Figure 5a, Figure S2a).

In contrast, AT-MSCs were able to express a high level of *Adipoq* and *Pparg* when compared to the CB-MSCs in adipogenic differentiation (over 4-fold for both genes) (Figure 5b and Figure S2b). The expression of adipogenic markers was less pronounced between different passages of MSCs. We also noticed that the expression of *Sox9* and *Col2a1* was high in both types of differentiated MSCs (Figure 5c and Figure S2c), which implies comparable chondrogenic differentiation efficiency. Taken together, the differential expressions of osteogenic and adipogenic markers are in agreement with the Alizarin red and Oil Red O staining patterns (Figure 4), suggesting that MSCs, although harboring multi-lineage differentiation potential, have a preference to differentiate towards their tissues of origin.

### 3.4. Inhibition of DNA Methylation Alters MSC Multipotency

**Figure 6:**
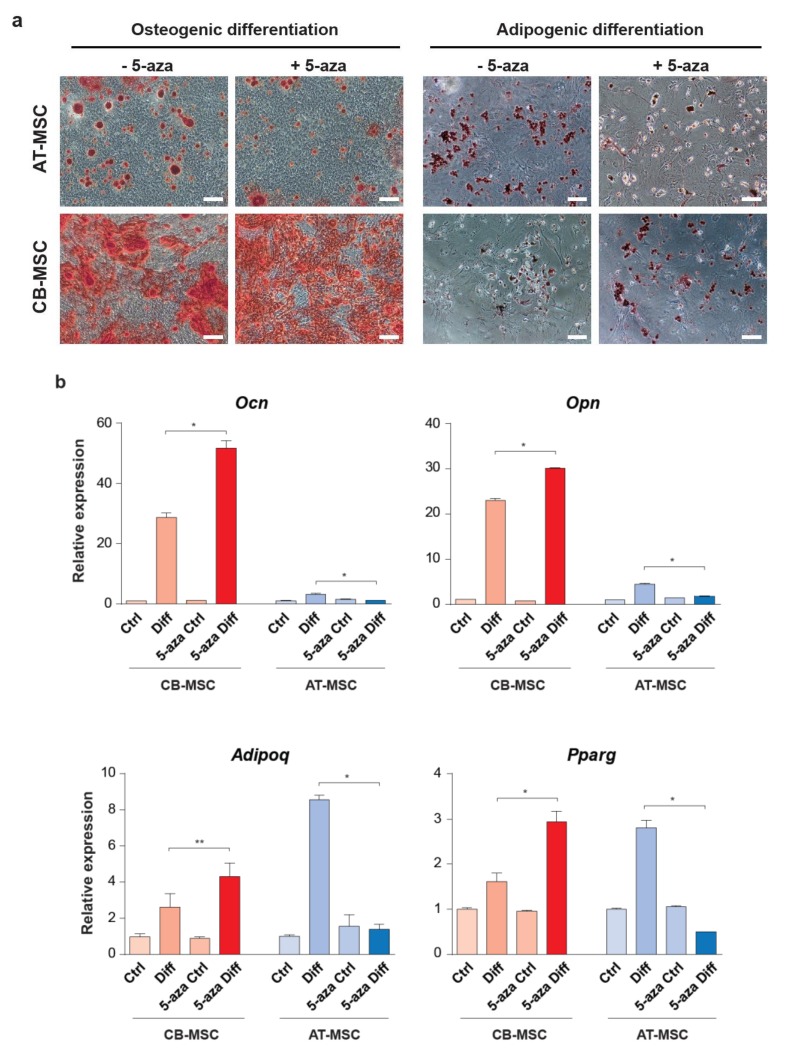
Osteogenic and adipogenic differentiation of the 5-aza-treated MSCs. (**a**) AT-MSCs and CB-MSCs were pre-treated with 5-aza for 48 h prior to osteogenic (Alizarin red staining) or adipogenic differentiation (Oil Red O staining). Representative images were taken at 20×. Scale bars: 100 µm. (**b**) The expression of osteocyte markers (*Ocn* and *Opn*) and adipocyte markers (*Adipoq* and *Pparg*) were determined by qRT-PCR. MSCs cultured in basic medium without differentiation agents for the same period of time were served as controls. Gene expressions were normalized with housekeeping gene *Gapdh*. Experiments were performed with three replicates. Data represent mean ± SD; * *p* < 0.05 and ** *p* < 0.01.

MSCs derived from different tissues could be modulated by the microenvironment which confers a differential epigenetic state associated with stem cell multipotency. To determine whether DNA methylation, a well-known epigenetic modification, is involved in the differentiation bias of CB-MSC and AT-MSC, we treated MSCs with the DNA methylation inhibitor, 5-azacytidine (5-aza), for 48 h prior in vitro differentiation. The 48-h treatment period was chosen based on the rationale that the epigenetic function of 5-aza as a DNA methylation inhibitor is dependent on cell division [30], which takes roughly 2 days (determined by the doubling time in Figure 2c) for both types of MSCs. Both types of MSCs under 5-aza treatment were able to differentiate into osteogenic and adipogenic lineages (Figure 6a). Interestingly, we observed that there were more osteogenic differentiated cells stained with Alizarin red from the 5-aza-treated CB-MSCs when comparing to the untreated sample. However, the number of osteogenic differentiated AT-MSCs remains low by the 5-aza treatment. This observation is in agreement with the qRT-PCR results of the osteogenic marker expression, which showed a significant increase in *Ocn* and *Opn* expression in the 5-aza-treated CB-MSCs (Figure 6b and Figure S3a). Unexpectedly, although the AT-MSCs are more competent to undergo adipogenic differentiation, the 5-aza treatment resulted in a lower number of Oil Red O stained cells (Figure 6a), with a significant decrease in the expression of adipogenic markers *Adipoq* and *Pparg* (Figure 6b and Figure S3b). By contrast, the treated CB-MSCs showed enhanced adipogenic differentiation with a comparable level of adipogenic marker gene expression to the untreated AT-MSCs. These results suggest that inhibition of DNA methylation can restore the biased differentiation capacity of CB-MSC to the adipogenic lineage, whereas AT-MSC loses its multipotency under the same epigenetic condition. It thus implies a differential epigenetic effect of 5-aza on the MSCs derived from different tissue sources.

## 4. Discussion

In this study, murine MSCs were isolated from compact bone and adipose tissue. Our results showed that prolonged culture of MSC leads to changes in cell morphology and cell surface marker patterns, and cell proliferation rate. Importantly, the tissue origins of MSC have impact on their differentiation capacity towards the corresponding cell lineages, indicating the presence of epigenetic memory in the MSCs. The multipotency of CB-MSC, but not AT-MSC, can be enhanced through inhibition of DNA methylation prior differentiation, which suggests a possible strategy to erase the epigenetic memory in certain tissue-derived MSCs. Although we were using murine MSCs in our study, cross-species comparisons of MSC corroborated that the surface markers [31] and biological functions [32] of MSC are similar, even though not identical, between different species. There are numerous studies of the therapeutic applications of MSCs using mouse models for the investigation of the molecular mechanisms and the safety concerns prior to human clinical trials [33]. With the findings of preferential differentiation of murine MSCs derived from different tissues and the possible manipulation of the epigenetic memory in MSCs, we propose that our findings can be applied to human MSCs for the selection of optimal tissue source and the strategy to enhance the multipotency of human MSCs for therapeutic applications.

The immunophenotype of MSCs is dynamic over the culture period. MSCs isolated from various tissues were reported to express a common set of cell surface markers, such as CD105, CD90, CD73, CD29, and CD44, with a lack of CD34, CD45, CD11b, and major histocompatibility complex (MHC) class II expression [25]. The MSC isolation protocols retained the plastic adherent cells from the tissue, which invariably consist of a heterogeneous cell population including hematopoietic cells and other tissue cells. Nevertheless, the non-MSC populations presumably undergo depletion gradually in the MSC culture condition, resulting in a more homogeneous MSC population. In our study, we observed that nearly all the early passage MSCs from both compact bone and adipose tissue expressed CD29 and Sca-1, but only 34–50% are CD105^+^ and 38–60% are CD106^+^, suggesting the heterogeneity of MSC immunophenotypes. Prolonged culture of MSCs leads to loss of Sca-1 and CD105 expression, which is particularly obvious in the AT-MSCs and CB-MSCs, respectively. Similar observation for the loss of CD15, CD90, and CD309 was also reported in the neoplastic transformation of bone marrow-derived MSCs after numerous passages [34]. The loss of MSC immunophenotype is correlated with the findings of fibroblastoid morphology change, reduced cell proliferation and differentiation potentials of the late passage MSCs, suggesting that the “stemness” of MSCs cannot be maintained by prolonged culture. This is in agreement with the previous study of the prolonged culture of human bone marrow-derived MSCs with a loss of osteogenic potential [35]. However, the loss of stemness could be less prominent in other tissue-derived MSCs, for example, umbilical cord MSCs, which retained comparable growth rate and osteogenic capacity after 16 passages when compared to the freshly isolated one [36]. Although the MSC culture condition used in this study follows a common protocol in the field, the alterations of MSC phenotypes and cell functions owing to the prolonged culture might reflect a suboptimal condition that needs to be further optimized for better maintenance of the stemness of MSCs. In addition, it has been reported that a subpopulation of MSC is CD105 negative [37,38], which varies in the differentiation potentials and modulation of CD4^+^ T cell proliferation when comparing to the CD105^+^ counterpart. Interestingly, the CD105 expression in the CD105^+^ MSCs can be altered by the culture condition, such as passage number, cell density, and medium composition [37,39]. Being a component of the TGF-β receptor, CD105 also serves as a proliferation marker of endothelial cells [40]. As we observed a reduction of MSC proliferation upon prolonged culturing, we speculate that this could be associated with the loss of CD105 expression in the P7 MSCs.

The biased differentiation capacity of MSCs derived from different tissues remains controversial. While several studies have reported that bone marrow-derived MSCs were more prone to osteogenic differentiation [17,41,42] and adipose tissue-derived MSCs showed decreased chondrogenic differentiation capacity [43,44], others demonstrated no significant differences in differentiation potentials of MSCs derived from various tissues [16,17,18]. In our study, we observed that both types of MSCs showed comparable chondrogenic differentiation, but preferential differentiation of AT-MSCs to adipo-lineage and CB-MSCs to osteo-lineage. It is proposed that the isolation procedures, culture condition, such as with serum or serum-free, and the heterogeneity of MSC populations may account for the preferential differentiation to certain lineages through enrichment of distinct MSC subpopulations. In addition, MSCs resided in different tissues are subjected to the distinct cellular microenvironments, e.g., signaling molecules, extracellular matrix components, metabolites, etc. These extrinsic factors can induce alterations of the MSC epigenome, leading to potential variations in transcriptomes and biological responses related to stemness [45]. This is indeed supported by the global transcriptomic and proteomic studies which demonstrated substantial differences in the expression of genes or proteins between MSCs derived from bone marrow and adipose tissue [17,20,46,47]. The biased differentiation of CB-MSC and AT-MSC also suggests that the altered epigenome is retained as the “epigenetic memory” of tissue origin. It is reported that the reprogrammed stem cells showed preferential differentiation towards their somatic lineage origins. The epigenetic memory of donor cell origin was found in *Xenopus* nuclear transplanted embryos [48] and early passage of induced pluripotent stem cells (iPSCs) [49,50]. It has been shown that blood-derived iPSCs were preferentially differentiated towards blood lineages and were defective to osteogenic differentiation, whereas bone marrow-derived iPSC demonstrated the opposite [50]. Although the derivation of MSCs does not involve cellular reprogramming, MSCs located at different tissues might be epigenetically reprogrammed by the tissue niche environment. Previous studies reported that the memory status in iPSC can be erased by extensive cell culture passages [49] or by epigenetic modifying agents, such as DNA methylation inhibitor [50]. It remains a challenge to test if the memory status of MSCs can be erased by prolonged culture because late passage MSCs undergo senescence and reduced overall differentiation capacity. Interestingly, our data showed that inhibition of DNA methylation can partially restore the adipogenic differentiation capacity of CB-MSCs, suggesting a possible way to erase the epigenetic memory in MSCs. However, we noticed that AT-MSCs failed to restore osteogenic differentiation after 5-aza treatment, suggesting the involvement of other types of epigenetic modifications, e.g., histone protein methylation or acetylation. Therefore, it is worth evaluating the effects of other epigenetic inhibitors, such as histone deacetylase inhibitors, on the multipotency of MSCs derived from other tissues.

## 5. Conclusions

We have demonstrated that early passage MSCs derived from compact bone or adipose tissue are highly proliferative and retain multipotent nature. The tissue origin of the MSCs results in epigenetic memory which implicates a preference for lineage differentiation. A better understanding of the molecular nature of such tissue origin memory can facilitate the choice of optimal sources of MSCs for tissue engineering and regenerative medicine.

## Figures and Tables

**Table 1:** Percentage of cell populations in AT-MSC and CB-MSC.

Sample	Passage	CD29	CD44	CD105	CD106	Sca-1	c-kit	CD11b	CD45
**AT-MSC**	**P3**	99.8 ± 0.1	18.3 ± 1.3	50.1 ± 2.4	38.1 ± 3.2	98.5 ± 0.7	2.1 ± 0.6	0.99 ± 0.2	0.71 ± 0.2
	**P7**	99.9 ± 0.0	41.8 ± 3.4 *	33.6 ± 4.3 *	54.3 ± 3.6 *	26.3 ± 4.4 **	1.8 ± 1.0	0.11 ± 0.1 *	0.1 ± 0.0 *
**CB-MSC**	**P3**	89.6 ± 4.2	87.9 ± 2.6	34.2 ± 3.3	60.2 ± 4.1	83.9 ± 5.4	6.7 ± 1.3	0.84 ± 0.4	38.4 ± 3.6
	**P7**	99.9 ± 0.1	94.5 ± 2.7	7.5 ± 1.7 *	99.1 ± 0.5 **	97.0 ± 1.8 *	0.3 ± 0.2 *	0.51 ± 0.3	1.1 ± 0.3 *

* *p* < 0.05 and ** *p* < 0.01 in the comparison between P3 and P7.

## Supplementary Materials

The following are available online at https://www.mdpi.com/2073-4409/9/3/756/s1, Figure S1: Immunophenotypes of hematopoietic markers on MSCs, Figure S2: Expression of lineage marker genes in the differentiated MSCs with reference to *β*-Actin gene, Figure S3: Expression of lineage marker genes in the differentiated 5-aza-treated MSCs with reference to *β*-Actin gene, Table S1: Primer sequences for qRT-PCR.
