# Supplementary material for: Murine Mesenchymal Stromal Cells Retain Biased Differentiation Plasticity Towards Their Tissue of Origin
Source: Cells. 2020 Mar 19;9(3):756. doi: 10.3390/cells9030756 (PMC7140683; doi:10.3390/cells9030756)
Supplement: Supplementary file 1 [file cells-09-00756-s001.zip › Supp Figure S1.pdf]

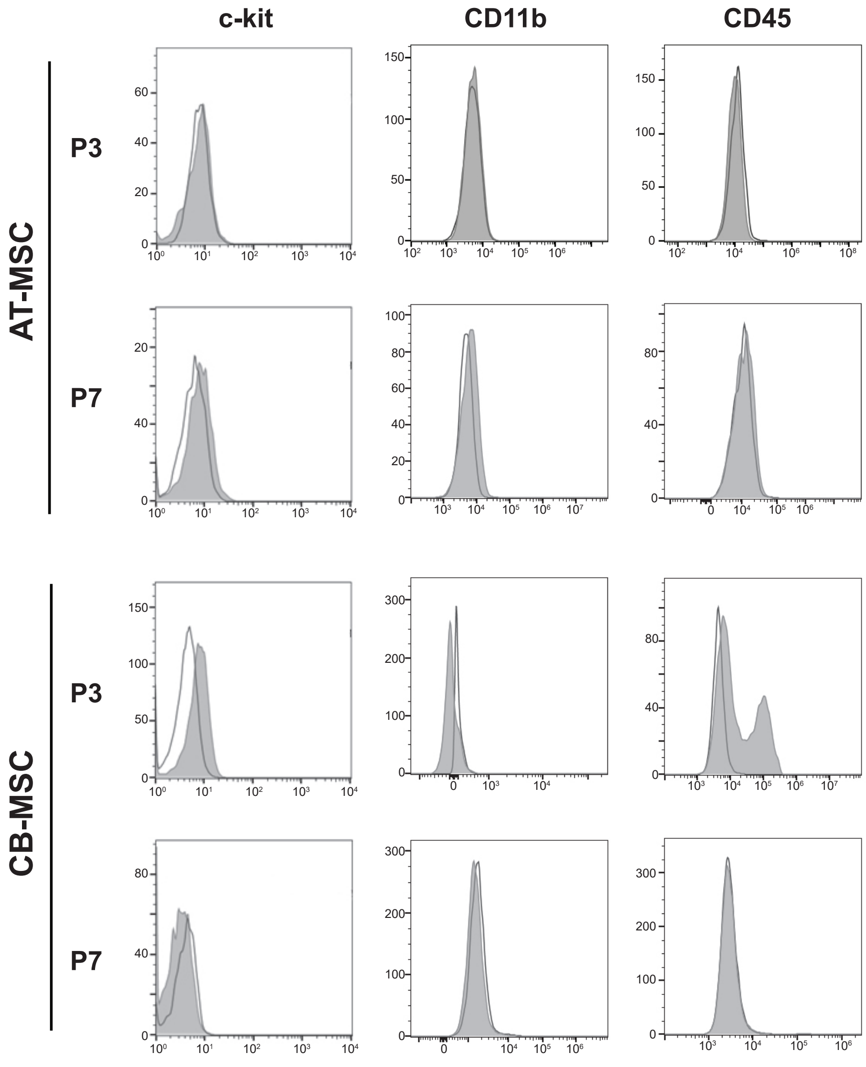

Figure S1. Immunophenotypes of hematopoietic markers on MSCs. Cell surface markers, c-kit, CD11b, and CD45, were used to characterize AT-MSC and CB-MSC at passage 3 (P3) and 7 (P7), respectively. Representative flow cytometry patterns were shown. Shaded peaks represent antibody-labeled population; blank peaks represented isotype controls.
