# Supplementary material for: Murine Mesenchymal Stromal Cells Retain Biased Differentiation Plasticity Towards Their Tissue of Origin
Source: Cells. 2020 Mar 19;9(3):756. doi: 10.3390/cells9030756 (PMC7140683; doi:10.3390/cells9030756)
Supplement: Supplementary file 1 [file cells-09-00756-s001.zip › Supp Figure S2.pdf]

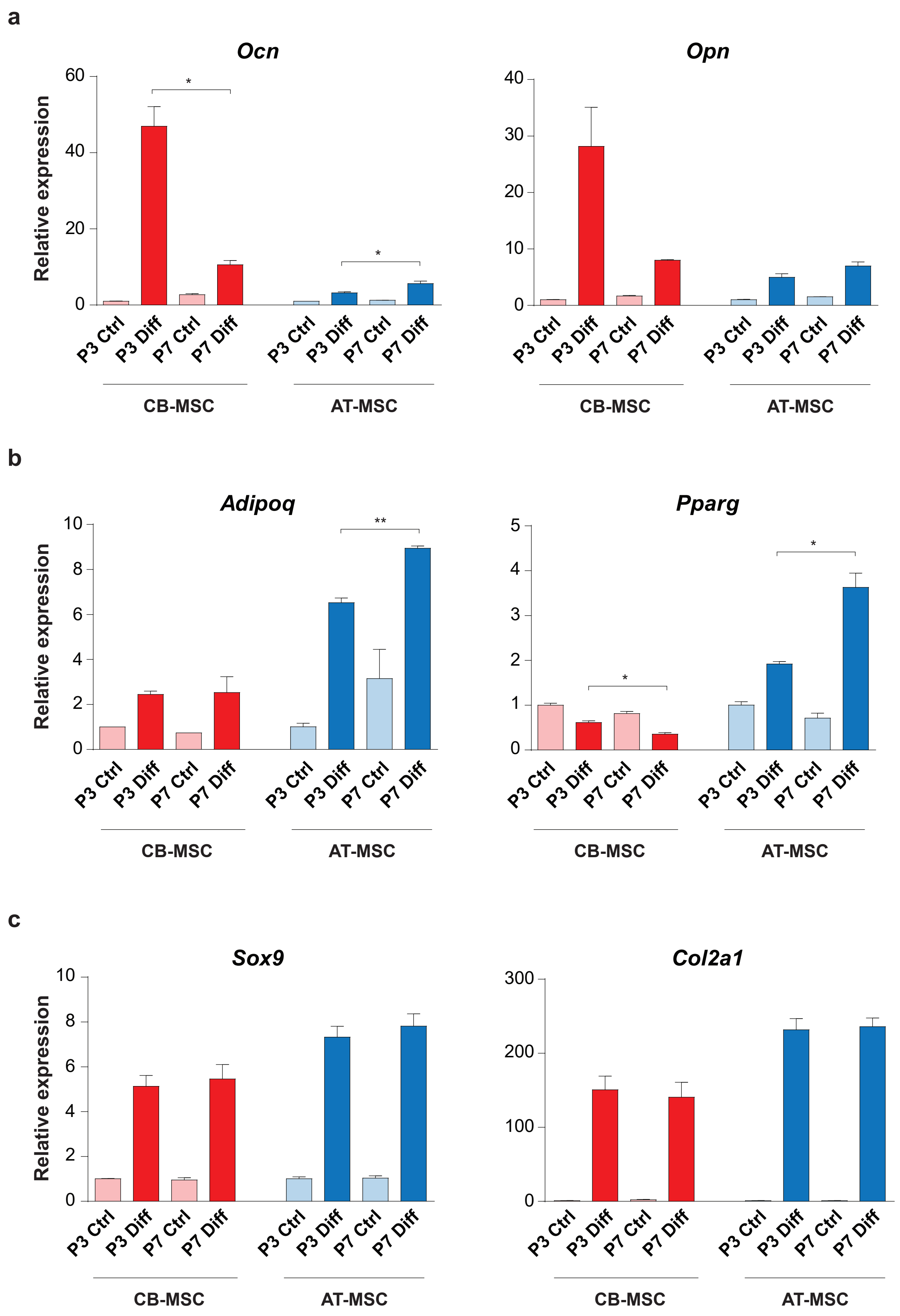

Figure S2. Expression of lineage marker genes in the differentiated MSCs with reference to β-*Actin* gene. **(a)** Osteogenic markers, *Ocn* and *Opn*, **(b)** adipogenic markers, *Adipoq* and *Pparg*, and **(c)** chondrogenic markers, *Sox9* and *Col2a1*, were used to determine the multi-lineage differentiation of AT-MSCs and CB-MSCs. MSCs cultured in basic medium without differentiation agents for the same period of time were served as controls. Gene expressions were normalized with housekeeping gene β-*Actin*. Experiments were performed with three replicates. Data represent mean ± SD; *$p < 0.05$ and ** $p < 0.01$.
