# Supplementary material for: Murine Mesenchymal Stromal Cells Retain Biased Differentiation Plasticity Towards Their Tissue of Origin
Source: Cells. 2020 Mar 19;9(3):756. doi: 10.3390/cells9030756 (PMC7140683; doi:10.3390/cells9030756)
Supplement: Supplementary file 1 [file cells-09-00756-s001.zip › Supp Figure S3.pdf]

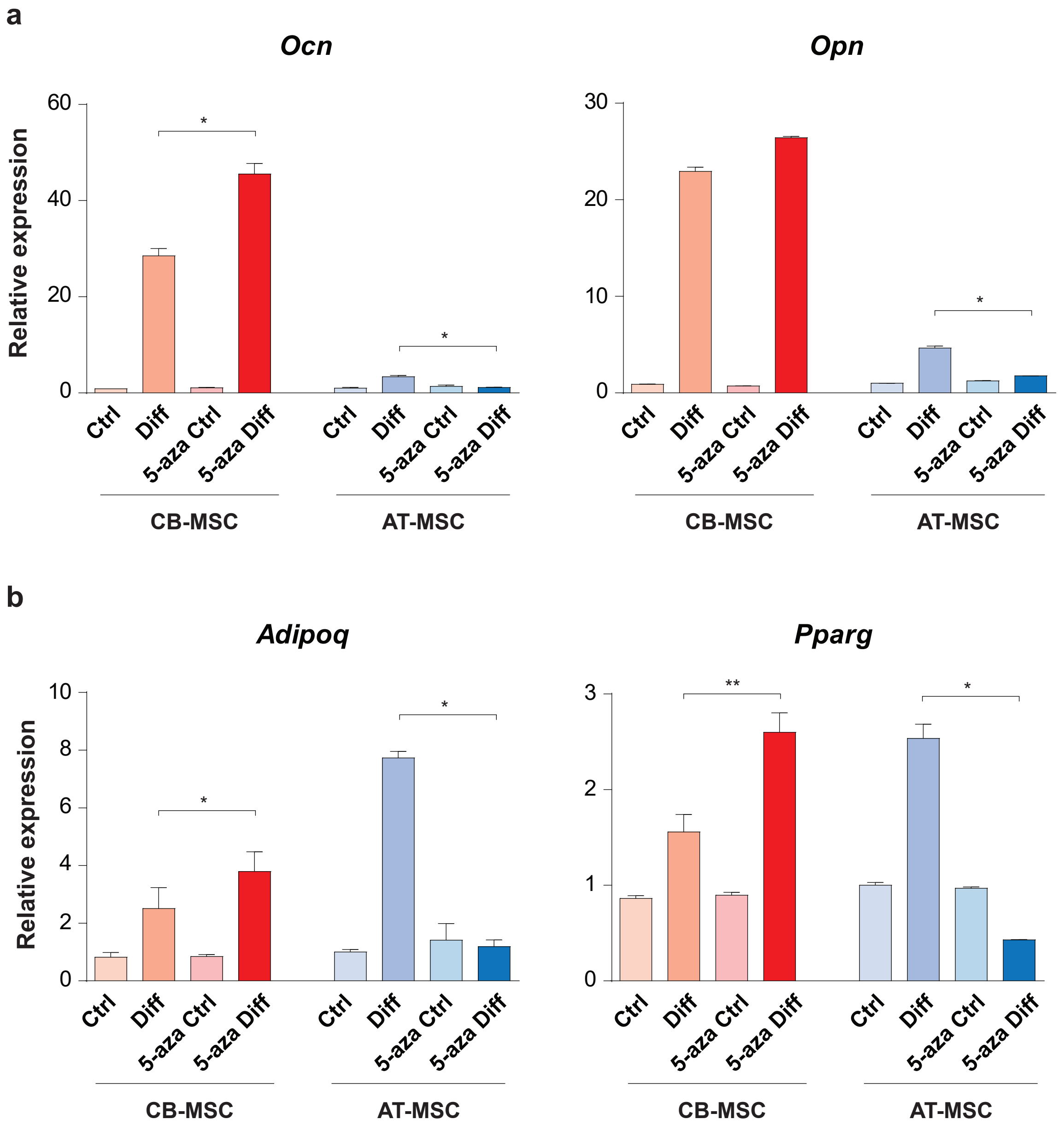

Figure S3. Expression of lineage marker genes in the differentiated 5-aza-treated MSCs with reference to β-*Actin* gene. The expression of **(a)** osteocyte markers (*Ocn* and *Opn*) and **(b)** adipocyte markers (*Adipoq* and *Pparg*) were determined by qRT-PCR. MSCs cultured in basic medium without differentiation agents for the same period of time were served as controls. Gene expressions were normalized with housekeeping gene β-*Actin*. Experiments were performed with three replicates. Data represent mean ± SD; *$p < 0.05$ and ** $p < 0.01$.
