# Supplementary material for: Murine Mesenchymal Stromal Cells Retain Biased Differentiation Plasticity Towards Their Tissue of Origin
Source: Cells. 2020 Mar 19;9(3):756. doi: 10.3390/cells9030756 (PMC7140683; doi:10.3390/cells9030756)
Supplement: Supplementary file 1 [file cells-09-00756-s001.zip › Supp Table S1.doc]

**Table S1.** Primer sequences for qRT-PCR.

| **Gene** | **Forward primer (5’ – 3’)** | **Reverse primer (5’ – 3’)** |
| --- | --- | --- |
| *Ocn* | CATGAGGACCATCTTTCTGC | CTTGGCATCTGTGAGGTCAG |
| *Opn* | AGGAAACCAGCCAAGGTAAG | TGCCAATCTCATGGTCGTAG |
| *Adipoq* | GGCCACTTTCTCCTCATTTC | AACAGGAGAGCTTGCAACAG |
| *Pparg* | GGATAAAGCATCAGGCTTCC | TCAATCGGATGGTTCTTCG |
| *Sox9* | AGAACAAGCCACACGTCAAG | CAGCAGCCTCCAGAGCTT |
| *Col2a1* | TATGGAAGCCCTCATCTTGC | CAGATAATGTCATCGCAGAGG |
| *Gapdh* | CAAGCTCATTTCCTGGTATG | TCTTGCTCAGTGTCCTTGCT |
| *β-Actin* | ACTATTGGCAACGAGCGGTT | ACACTTCATGATGGAATTGAATGTAGT |
